# Self-powered wearable electronics

**DOI:** 10.1017/wtc.2020.3

**Published:** 2020-10-13

**Authors:** Puchuan Tan, Yang Zou, Yubo Fan, Zhou Li

**Affiliations:** 1 Beijing Advanced Innovation Centre for Biomedical Engineering, Key Laboratory for Biomechanics and Mechanobiology of Chinese, Education Ministry, School of Biological Science and Medical Engineering, Beihang University, Beijing, China; 2 CAS Center for Excellence in Nanoscience, Beijing Key Laboratory of Micro-Nano Energy and Sensor, Beijing Institute of Nanoenergy and Nanosystems, Chinese Academy of Sciences, Beijing, China; 3 Center on Nanoenergy Research, School of Physical Science and Technology, Guangxi University, Nanning, China

**Keywords:** bioelectronics, nanogenerator, self-powered, wearable electronics

## Abstract

Wearable electronics are an essential direction for the future development of smart wearables. Among them, the battery life of wearable electronics is a key technology that limits their development. The proposal of self-powered wearable electronics (SWE) provides a promising solution to the problem of long-term stable working of wearable electronics. This review has made a comprehensive summary and analysis of recent advances on SWE from the perspectives of energy, materials, and ergonomics methods. At the same time, some representative research work was introduced in detail. SWE can be divided into energy type SWE and sensor type SWE according to their working types. Both types of SWE are broadly applied in human–machine interaction, motion information monitoring, diagnostics, and therapy systems. Finally, this article summarizes the existing bottlenecks of SWE, and predicts the future development direction of SWE.

## 1. Introduction

Wearable devices have evolved to this day, with more diverse forms and more abundant functions. At the same time, users’ requirements for wearable electronics are becoming more strict, and these requirements contain miniaturization, multifunction, and intelligence. Among all the requirements, one of the most important requirements which influence the user experience is the battery life of wearable electronics.

In most research and production, wearable devices were powered by batteries. However, due to the limitations of the battery industry, batteries themselves often occupy most of the space and weight of wearable devices, which go against the portability of wearables (Zheng et al., 2016). The invention of the lithium battery has revolutionary significance in the development of the battery industry (Wang et al., 2019b). Its characteristics, such as lightweight, low pollution, safety, and high specific energy are in line with the relevant requirements of wearable devices, which have greatly promoted the development of wearable devices. However, even a large-capacity lithium battery will eventually run out of energy, which cannot avoid the trouble of repeated charging (Yu et al., 2013). Especially with the development of wearable devices, the energy consumption of the load part gets higher and higher. Therefore, to provide a long-time and reliable energy source, it is necessary to develop a self-powered wearable device.

In response to the need of society, self-powered wearable electronics (SWE) have emerged. Researchers have begun to try to collect various forms of energy surrounding the human body by different kinds of generators, designed them into different types of self-powered systems, and applied them in a variety of applications. In much work, researchers chose mature power generation technologies to provide energy sources for SWE, such as solar cells (Zhang et al., 2014), electromagnetic generators (EMG) (Tan et al., 2019), and thermoelectric generators (TEG) (Zhang et al., 2019a). These power generation technologies have unique characteristics and are suitable for different occasions. However, the power generated by these power generation technologies is not sufficient to meet the requirements of long-term use of wearable electronics. Their power generation efficiency needs to be strengthened. The successive invention of piezoelectric nanogenerator (PENG) and triboelectric nanogenerator (TENG) has greatly promoted the research and development of SWE (Liu et al., 2017). They have high output voltage and high energy conversion rate, can collect small-scale mechanical energy, and respond to low-frequency mechanical motion (Jiang et al., 2016). Especially, the TENG has the advantages of simple production, a wide selection of materials, and it has broad application scenarios (Ouyang et al., 2017).

SWE systems are designed by collecting energy or information from the environment surrounding the human body. For collecting energy and for collecting information are two so different types of application purposes, we have to discuss them separately. SWE that collects energy around the environment and applies it to other wearable devices, we call it energy type SWE, and SWE that collects information around the human is the sensor type SWE. Both of them have a wide range of essential applications in the fields of biomedicine, military, wireless communications, and wireless sensing (Khaligh et al., 2010; Zang et al., 2015).

In some previous review papers, Lou et al. discussed the research progress of sensor type SWE (Lou et al., 2017). Wu and Haick introduced the current research status of SWE in biomedical sensing from the perspective of materials (Wu and Haick, 2018). Liu et al. focused on SWEs based on TENG and discussed the applications of these SWEs in the biomedical field (Liu et al., 2019a). These papers provide a good guide for researchers in their respective fields. Still, the scope and focus of our review are different. This review sorts out much work in the SWE field and selects some representative work from different application directions. Through these representative works, readers can systematically understand the research progress, bottlenecks, and possible solutions of specific research directions. Meanwhile, this review also summarizes the materials and ergonomics methods selected by most of SWEs. At last, this review discusses and prospects the existing problems, possible solutions, and future development direction in the entire SWE field.

## 2. Energy

As self-powered electronics, the first requirement is to solve the problem of energy supply. SWE does not require an additional power source, and its power comes from other forms of energy in its working environment.

### 2.1. Energy resource

**Figure 1.**
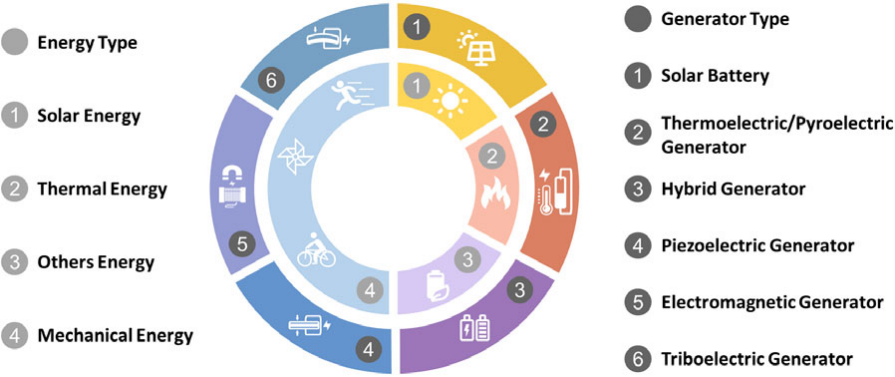
Various energies surrounding human live environment (inner circle) and generators that gather these energies (outer circle).

There are many forms of energy around the human body, including light energy, thermal energy, mechanical energy, and so on (Zheng et al., 2018). To collect this energy, different generators based on different principles are necessary (Figure 1).

#### 2.1.1. Solar energy

Solar energy is a renewable and clean energy source, and humans have widely used it for a long time. Solar cells commonly use various semiconductor materials, and they are light and convenient to carry (Zhang et al., 2014). The power generation principle of most such power generation equipment is based on the photoelectric effect (Kim et al., 2018). The photoelectric effect is the emission of electrons or other free carriers when light hits a material (Jinno et al., 2017). Electrons emitted in this manner can be known as photoelectrons. By photoelectric effect, light energy is directly converted into electrical energy and used as an energy source for wearable electronics (Feng et al., 2019). Generally, in our living environment, the sunlight is relatively sufficient, which can ensure the charging demand of SWE using solar energy.

#### 2.1.2. Thermal energy

Thermal energy is another common form of energy in nature, and the human body as a heat source can be employed as an energy source for devices based on thermoelectric effects (Du et al., 2015). The effect of thermoelectric power generation is based on the Seebeck effect. The Seebeck effect is the build-up of an electric potential across a temperature gradient (Shen et al., 2018). A thermocouple measures the difference in potential across a hot and cold end for two dissimilar materials. This potential difference is proportional to the temperature difference between the hot and cold ends. Common inorganic thermoelectric materials are usually brittle and fragile, and they cannot meet the requirements of current wearable electronics for the flexibility and stretchability of devices. In response to this problem, more and more 2D thermoelectric materials with excellent performance have been developed. Another essential issue of thermoelectric power generation is the problem of energy collection efficiency. Skin thermal resistance, material thermal resistance, and thermal diffusion are the possible reasons for limiting energy collection efficiency (Feng et al., 2019). Therefore, more efficient thermoelectric materials and structures need to be developed.

#### 2.1.3. Mechanical energy

Various movements of the human body can often produce a large amount of mechanical energy (Shi et al., 2019). If this mechanical energy could be utilized correctly, they can also act as the energy supply of self-powered equipment. Currently, conventional devices for converting mechanical energy into electrical energy include EMG, PENG, and TENG.

The EMG is a mature power generation device, and it is also the main power generation equipment in human society. The principle of EMG is based on Lenz’s law, that is, the current induced in a conductor by a changing magnetic field. However, the output of an EMG and the motion frequency is highly relevant (Zi and Wang, 2017). The human body’s motion frequency is generally 1–5 Hz, which belongs to low-frequency motion. Therefore, for EMGs, the mechanical energy generated by the human body’s motion is not as abundant as other generators. Complex mechanical structures are often needed to make up for this problem, which increases the difficulty of design and also challenges the size requirements of miniaturization. At present, EMG is uncommon in SWE as a single generator system. They are often combined with some other power generation forms to make hybrid generators to drive SWE.

Researchers have discovered the piezoelectric effect for a long time. Until now, it has been extensively used in wearable electronics. The piezoelectric effect is the ability of certain materials to generate an electric charge in response to applied mechanical stress. Based on this phenomenon, Zhong Lin Wang proposed the PENG (Amjadi et al., 2014). In the Early time, PENG often used some brittle inorganic piezoelectric materials, which were not suitable for the field of wearable electronics. With the deepening of research, new organic piezoelectric materials have been developed, and piezoelectric materials have begun to become flexible and thin. It also makes PENG more suitable for SWE.

The TENG proposed by Zhong Lin Wang in 2014 is giant step forward in the field of energy. The principle of TENG is based on the coupling of friction electrification and electrostatic induction (Chen et al., 2018a, 2018b). The wide selection of materials for TENG has greatly promoted its vigorous development in various fields. On the other hand, TENG and PENG also have a good ability to collect mechanical energy in low-frequency conditions, which make these two types of generators well used in motion energy collection. TENG has four basic modes, namely contact-separated TENG, sliding TENG, single-electrode TENG, and free-standing TENG. All four TENGs have a wide range of applications in mechanical energy harvesting. TENG and PENG have high output voltages and relatively low currents and require additional circuits to match the current energy supply of wearable electronics.

#### 2.1.4. Other energy sources

Besides the various forms of generators referred to above, there are some power generation devices that use other principles to generate electricity, such as biofuel cells (Dagdeviren et al., 2017). All those that can utilize the energy of the surrounding environment of wearable electronic products to achieve self-powered energy supply should be considered as SWE.

Also, to achieve the purpose of increasing power output and increasing the scope of application, a hybrid generator is a universal idea. Hybrid generators often integrate two or more generators to reach the purpose of generating electricity (Leonov et al., 2009). At present, the core problems of hybrid generators include requirements for power matching, structural design, and electric circuit design. With the deepening of research, hybrid generators have shown increasing application prospects in the energy supply of SWE.

### 2.2. Energy management

After the energy has been gathered, it cannot be directly used by SWE. The energy needs to be processed by some energy management module before used. Before explaining the energy management module, wearable electronics need to be divided into two types according to their work types: one is energy-type SWE, and the other is sensor-type SWE. The two types of SWE have fundamental differences in energy management strategies. The energy management module is very important for SWE, and its research content involves the field of electronics. Generally speaking, under the condition of ensuring the normal processing of electric energy, the energy management module often needs to meet the requirements of miniaturization and high integration.

#### 2.2.1. Energy type SWE

**Figure 2.**
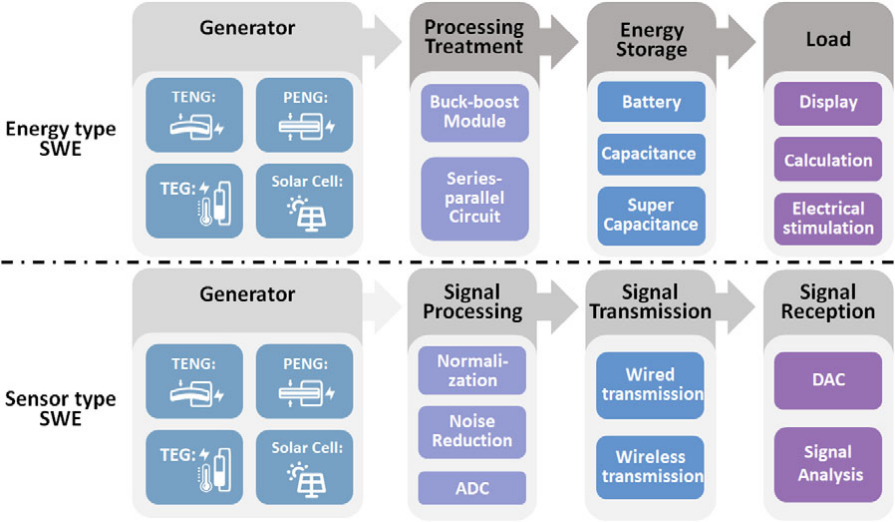
Energy management of energy type SWE and sensor type SWE. According to the difference in the form of energy utilization, SWE can be divided into energy type SWE and sensor type SWE. The energy management of energy type SWE is to process the energy, store the energy, and then supply energy to various loads. The energy management of sensor type SWE is to process the signal and transmit the signal to the receivers.

The main goal of this type of SWE is to collect various energies from the wearer’s surrounding environment, store it, and then apply it to the load module. Such as display and calculation or electrical stimulation. Therefore, the core research issues of energy type SWE in energy management strategies include power adaptation, power boost, and power storage (Figure 2).

The energy generated by the SWE’s energy collection module is different from alternating current (AC) and direct current (DC), and AC needs to be rectified to DC using a rectifier bridge before being used. Moreover, the energy, voltage, and current generated by the energy collection module often do not meet the requirements of the load module, and cannot be directly utilized. The voltage or current has to be adjusted to an appropriate size through a buck-boost module and then applied. The buck-boost module may adjust the voltage, but the increase of the current must bring the attenuation of the voltage, and the increase of the voltage must also accompany the attenuation of the current. To increase the current without changing the voltage, parallel the generators into a generator pack is a feasible idea. Similarly, series-connected generator packs are a realistic idea for boosting voltage without changing current.

After the electric power is processed, the electric power is generally stored through an energy storage device. Commonly used energy storage devices include lithium batteries, capacitors, and supercapacitors (Gao et al., 2018). These three types of energy storage devices are different and suitable for different application scenarios. Generally, the battery is ideal for a long-term stable DC power supply working mode, and the working time of the capacitor and the supercapacitor is relatively short, which is suitable for the working scenario that needs to release the electric stimulation. The main load of energy type SWE generally includes the display, calculation, or electrical stimulation.

#### 2.2.2. Sensor type SWE

Another important application area of SWE is to collect information. The electricity generated by the power generation module is both an energy source and a signal. The analysis of this electrical information can effectively realize the situation of the wearer’s surrounding environment and the wearer’s physiological characteristics. Sensor type SWE is entirely separate from the energy type SWE in the energy management strategy. Sensor type SWE does not have high requirements for output voltage, and it has no provision for current (Nguyen et al., 2015). Its research focuses on linearity, sensitivity, and response time of the device.

Because of the internal resistance of the generator, sensor type SWE often requires the adjustment of adaptive resistance, so that the output of the generator exhibits better linearity with physical quantity. On the other hand, for more effective and reliable information, normalization, noise reduction, and analog-to-digital converter are also conventional methods for sensor type SWE. Unlike energy type SWE, sensor type SWE does not require an energy storage module but a signal transmission module (Zhang et al., 2019c). The primary signal transmission method can be subdivided into the wired signal transmission and wireless signals. Finally, signals are summarized to the signal conception module for analysis and identification.

## 3. Materials

**Figure 3.**
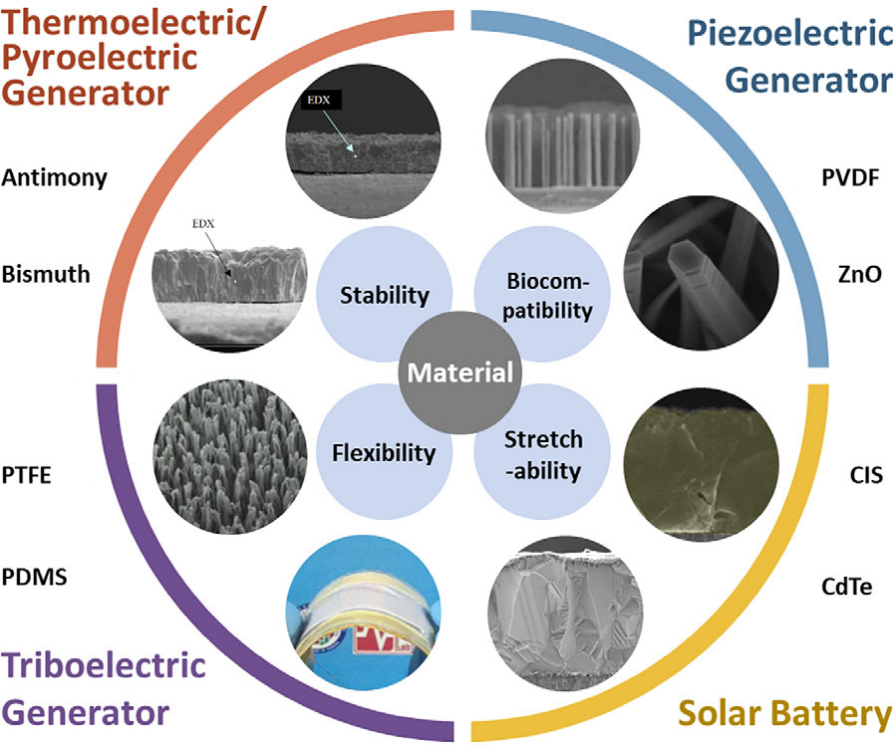
Materials used in SWE and their basic performance requirements. For each of the four different types of SWE, two typical materials are revealed here. Considering their application environment, stability, biocompatibility, flexibility, and stretchability of these materials need to be studied in detail.

In the previous content, we mentioned various types of generators used to collect energy. The power generation principles of these generators are different, and the choice of materials is also different. For TENG, polymer materials with strong electron withdrawing ability are often selected, such as polytetrafluoroethylene (PTFE) and polydimethylsiloxane (PDMS). For PENG, some materials with piezoelectric effect are needed, such as polyvinylidene fluoride (PVDF) and ZnO. Still, these generators are different in the choice of power generation materials, but because they have the same application scenario, they need to meet some same basic requirements. First, because SWE is applied to humans, the materials selected must be nontoxic and harmless and have excellent biocompatibility. At the same time, specific chemical stability must be met. SWE materials need to have good mechanical properties (Wu and Haick, 2018). Because it generally directly contacts human skin, and hard objects can affect the wearing experience. SWE often needs to be flexible and stretchable (Huynh and Haick, 2018). Because SWE is often used for a long time and the application scenario is complicated, the mechanical stability of the material is necessary to be considered when selected (Figure 3) (Cao et al., 2017). For energy type SWE, the conversion efficiency of material power generation needs to be considered, and for sensor type SWE, its sensitivity and response time need to be considered (Lin et al., 2017). Furthermore, some special functions of SWE also need to have some extra performance. For example, SWE applied in the electronic skin scene always needs to meet transparency requirements.

The materials are classified according to their functions in SWE, which can be divided into three categories. The first is the working material, critical material that converts other energy into electricity. Different principles of SWE have different working materials. For example, PVDF is often used as piezoelectric materials, and TEG often uses telluride and its alloys (Chen et al., 2018c). It is worth stressing that TENG has an extensive range of choices for its working materials (Table 1). In theory, as long as the two materials located between the triboelectric sequences can be used as working materials in TENG. The second is the electrode material. Because SWE often needs to meet the requirements of flexibility and stretchability, traditional metal electrodes are no longer applicable. Through some special processing or structural design, metal electrodes can become flexible and stretchable. Besides, the development of some flexible electrodes, such as ITO and hydrogel, has also provided more choices for SWE’s electrode materials (Lou et al., 2018). The third material is supporting material, and their role is to isolate, encapsulate, and protect the working part of SWE. Commonly utilized supporting materials are silicone, PDMS, and so on. Some materials can perform multiple functions. For example, PDMS can be used as working material in TENG or as supporting material.

**Table 1.** Material, principle, application, and performance of typical SWEs

Material	Type of SWE	Working mode	Operating voltage	Performance	References
Sb/Bi	Energy type SWE	TEG	0.25 V	Flexibleness/stability	Qu et al. (2001)
Perovskite /PEDOT	Energy type SWE	Solar cell	0.71 V	–	Xu et al. (2016)
PVDF	Energy type SWE	PENG	1.4 V	Efficiency	Cha et al. (2011)
PTFE/PDMS	Energy type SWE	TENG	23.5 V	Stretchability	Kwak et al. (2017)
PTFE/Ti	Energy type SWE	TENG	70 V	High efficiency	Zhao et al. (2019)
Cu/PZT/PTFE/Au	Energy type SWE	TENG/PENG/EMG	30 V	Universality	Tan et al. (2019)
Kapton/Cu	Sensor type SWE	TENG	1.52 V	Flexibleness/ultrasensitivity	Ouyang et al. (2017)
Graphene	Sensor type SWE	TEG	0.7 mV	Ultrasensitivity	Zhang et al. (2019b)
Enzyme/ZnO	Sensor type SWE	PENG	0.2 V	Flexibleness	Han et al. (2017)
PTFE/Ag	Sensor type SWE	PENG	1.613 V	Stretchability/ultrasensitivity	Amjadi et al. (2014)
PMN-PT/Au/PET	Sensor type SWE	PENG/TEG	1.5 V	Flexibleness	Chen et al. (2017)

## 4. Ergonomics

**Figure 4.**
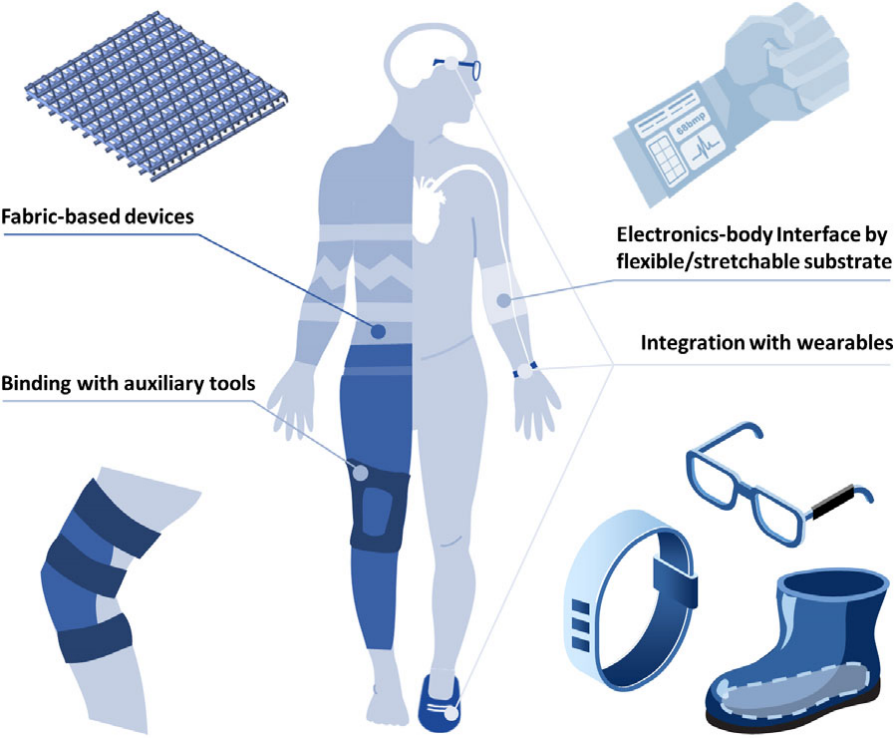
Four kinds of primary ergonomics methods of SWEs: fabricated fabric-based wearable devices; utilized flexible/stretchable substrate to construct an electronics–body interface; bound devices with auxiliary tools; integrated devices with wearables. By these four wearing strategies, SWEs can contact with the human body closely and firmly, and collect energy or signals generated by the human body effectively.

In the previous content, we have explained how SWE achieves the requirement of self-powered from the perspective of principles and materials. The other basic requirement of SWE is wearing. In order to realize the goal of wearing, there are four commonly used ergonomics methods: fabric-based device, human–machine interface by flexible/stretchable substrate, integration with wearables, and binding with auxiliary tools (Figure 4). Additionally, the rise of implanted medical devices has provided a long-time working idea for SWE (Liu et al., 2019a). Different ergonomics methods use different strategies to achieve wearability, and each has its own advantages and limitations. When discussing different wearing strategies, they need to be put together for comparison. At the same time, the scope of the application must be clarified according to their respective characteristics.

### 4.1. Fabric-based devices

Fabrics are a common type of wearables. Dope coating, dip dyeing, and other methods are used to dope effective working materials into existing product lines (Chai et al., 2016). Moreover, organic materials could be directly made into one-dimensional fabric by electrostatic spinning. Then, one-dimensional material is manufactured into a two-dimensional planar structure through complex and specially designed weaving means. The fabric obtained can possess the essential characteristics required by the two wearable electronic products, stretchability, and flexibility, through a special knotting design (Hong et al., 2019). Because the fabric can be worn directly on the surface of a person’s body, it has a large effective working area. At the same time, because both power generation materials and energy storage materials can be manufactured into fabric, fabric-based SWE can efficiently integrate these two modules. Because this method makes the material directly contact the skin, the most crucial issue that is required to be considered is the biocompatibility of the material. Besides, because of human sweating, fabric-based SWEs need to ensure a certain output under humid conditions (Zou et al., 2019). Another issue that demands to be considered is the mechanical stability of the material. On the one hand, the fabric requires a certain tensile strength of the material. On the other hand, due to the continuous stretching-recovery cycle, the material also needs to have marvelous mechanical stability.

### 4.2. Human–machine interface by flexible/stretchable substrate

A human–machine interface, or called electronic skin, is often made of a flexible substrate. Common flexible substrates include PDMS and silicone. The material is poured into a predesigned mold by inversion and taken out after curing. In order to realize functions such as sensing or energy collection, the flexible circuit is generally buried in a flexible substrate in advance and molded together. Due to the selection of organic polymer materials such as PDMS and silica gel, they often have the advantages of flexibility, stretchability, and transparency (Lou et al., 2017; Liu et al., 2019a). Since the human–machine interface made of a flexible substrate is also in direct contact with the skin, biocompatibility is also a requirement to be taken into account. Besides, although the device’s packaging material is flexible, the current commonly used circuits are rigid. The difference between Young’s module of two materials often causes material damage. In order to address this problem, correspondingly, the design and preparation of flexible circuits are also vital. Some hard parts, such as circuits, can comply with the requirements of flexibility and stretchability through the design of the serpentine structure. Even so, because some commercial electronics such as capacitors, resistors, and inductors are hard, full flexibility is still a significant problem and research direction for human–machine interfaces. Rogers and Bao’s group have outstanding research results in flexible electronics. Regardless of the fact that they have not directly studied SWE, their work can significantly inspire SWE researchers who make electronic skins. Human–machine interface by flexible/stretchable substrate requires precise circuit design and it has distinct advantages in array and integration. This character makes it more suitable for application to sensor-type SWE.

### 4.3. With wearables

With the maturity and popularity of existing smart bracelet technology, the strategy of integrating SWE into existing wearables has become more and more accepted. Besides integration on the bracelet, there is also some other work to integrate SWE into shoes or glasses, making smart shoes or smart glasses (Fan et al., 2016; Chen et al., 2017), and so on. The integration work is faced with more engineering problems. That is, how to incorporate the device into the existing wearable under limited space without affecting the functions of the SWE. Limited by size, the effective working area of smart glasses and smart bracelets is insufficient, and more application fields are focused on sensor type SWE (We et al., 2014). Smart shoes have a relatively larger working area, and mechanical energy generated by a human during moving is large. Thus, smart shoes can be applied in the field of energy collection. Additionally, since electronics are often integrated inside the wearables, there are no strict requirements on the choice of materials, but miniaturization and integration are necessary.

### 4.4. Binding with auxiliary tools

Using elastic bands to bind SWE to the body surface is a more general method, but it is also a relatively rough method. This method only uses auxiliary tools to fix the device to the body surface forcibly and does not form a suitable human–machine interface. The integration is not high, and it is not very convenient to wear. It often needs a long time to wear and fix. However, this method is still a universal method that can be used extensively. In the early stages of research, researchers can use this strategy as a temporary solution and then make reasonable improvements. In addition, when the entire device is sheet-like and has a certain flexibility, this method can still be considered for simple fixing (Chen et al., 2019).

## 5. Applications

Based on the various principles, materials and ergonomics discussed above, various SWEs for different scenarios have been developed. Some practical applications of SWEs have been introduced, especially as mentioned in the energy management section. Depending on the classification of energy use, the main research areas of SWE are focused on the energy and sensing field. The application object of SWE is human. The major search field of SWE is the self-powered wearable medical system and the self-powered wearable intelligent system. Especially in the medical field, SWE has a vast range of applications.

### 5.1. Self-powered wearable medical system

Modern medical service is becoming more and more digital, mobile, personal, and intelligent based on the application of the International of Things (IoT) technology and the rapid development of 5G communication technology (Hassanalieragh et al., 2015). Medical IoT serves the field of medical and health. It combines a variety of medical sensors and performs the information exchange and preliminary processing between mobile terminals, embedded computing devices and medical information processing platforms under the agreement through the sensor network (Yuehong et al., 2016; Zhu et al., 2019). The wearable medical electronic device plays an essential role in the perception layer of Medical IoT, which is an indispensable part of helping users obtain their health information and assisting with medical care (Haghi et al., 2017). The emergence of the self-powered wearable medical system has dramatically extended the lifespan of wearable medical devices, which enables both real-time and long-term health monitoring or disease diagnosis. Moreover, collecting energy from the human body for disease treatment also provides tremendous opportunities for telemedicine and long-term health care. In this section, we review the significant advances in self-powered wearable diagnostic and therapeutic systems research in recent years.

#### 5.1.1. Self-powered wearable diagnosis system

Self-powered wearable diagnosis systems monitor various physiological signals of the subjects through different principles. The monitoring content includes sweat, pulse, respiration, body temperature, and so on. Most of the self-powered wearable diagnosis system belongs to sensor type SWE. The self-powered wearable diagnosis system has huge market demand and is a research hotspot in the field of SWE.

##### 5.1.1.1. Self-powered sweat monitor system

Sweat is the fluid secreted by the sweat glands of the human body. In addition to 99% of the water in each drop of sweat, it also contains various ions, amino acids, hormones, proteins, peptides, and other secretions (Jadoon et al., 2015). People can monitor the degree of electrolyte imbalance, lactic acid index, sweat glucose levels, dehydration, and calories burned from the sweat. Related research demonstrates that health indicators in sweat are almost as plentiful as blood (Bariya et al., 2018). Blood tests usually require blood drawing, while sweat tests can be done outside the human body, which is noninvasive and painless. Therefore, sweat tests are called upon to become a more convenient test method than blood tests. Besides, due to its simple detection process, low detection cost, and low power consumption, it is also effortless to integrate with wearable devices.

**Figure 5.**
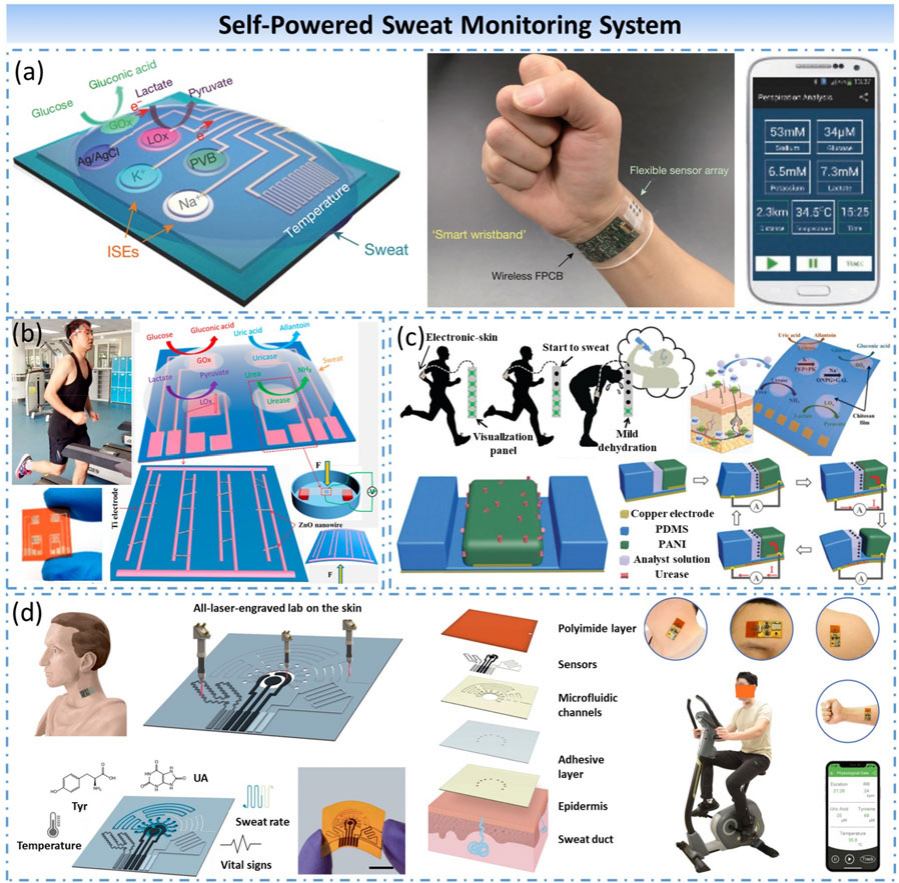
Self-powered sweat monitor system. (a) Multiplexed in situ perspiration analysis system based on a flexible integrated sensing array. (b) Noninvasive electronic-skin based on piezo-biosensing unit matrix. (c) Self-powered electronic-skin based on triboelectric-biosensing unit matrix. (d) Uric acid monitoring system based on an entirely laser-engraved wearable sensor.

In 2016, Gao et al. developed a wearable flexible integrated sensing array for multiplexed in situ perspiration analysis (Gao et al., 2016) (Figure 5a). This highly integrated flexible sensor array system can simultaneously and selectively detect the concentrations of glucose, lactic acid, sodium ions, and potassium ions in sweat, and wirelessly transmit the detection results to a smartphone without any external analysis and external circuit. In 2017, Han et al. demonstrated a piezo-biosensing unit matrix of enzyme/ZnO nanoarrays, which can be attached on the forehead of a runner to detect lactate, glucose, uric acid, and urea in the perspiration (Han et al., 2017) (Figure 5b). Piezoelectric output of ZnO nanoarrays modified by the different enzymes can reflect the concentration of different target analytes, with no needing for external power supply. In 2018, He et al. presented a self-powered electronic-skin based on polyaniline triboelectric-biosensing unit matrix for real-time perspiration analysis (He et al., 2018) (Figure 5c). The self-powered e-skin combines the triboelectric effect with enzymatic-reaction, which can convert the mechanical energy of human movement into electrical energy and used it to detect the concentration of different biomarkers. In 2020, Yang et al. proposed an entirely laser-engraved wearable sensor (Yang et al., 2020) (Figure 5d). The graphene-based chemical sensor system enables continuous wireless monitoring of low concentrations of uric acid and tyrosine in sweat simultaneously. The system was designed to assess uric acid levels in the sweat of gout patients and healthy subjects, and the results showed the same trend as uric acid levels in serum, which indicated a promising approach for personalized monitoring of UA levels.

##### 5.1.1.2. Self-powered body temperature monitor system

Body temperature is an essential vital sign of the human body. The body temperature of healthy people is relatively constant. When the body temperature deviates from the normal body temperature, it will affect the regular operation of the body’s metabolism and cause disorders in the functions of various cells, tissues, and organs. Therefore, body temperature monitoring is of considerable significance to human health assessment and can be utilized for the prevention and diagnosis of clinical diseases. Traditional body temperature measurement methods are usually single-point measurements. In recent years, some studies have shown that continuous and dynamic body temperature monitoring has higher medical value than single-point measurement for temperature diagnostics, which means a new perspective on the regulation and characteristics of the disease (Li et al., 2017). The development of continuous dynamic body temperature monitoring is inextricably linked to the support of wearable devices and self-powered technology. Therefore, it is precious to develop a self-powered wearable device that can continuously monitor the body temperature condition all day.

**Figure 6.**
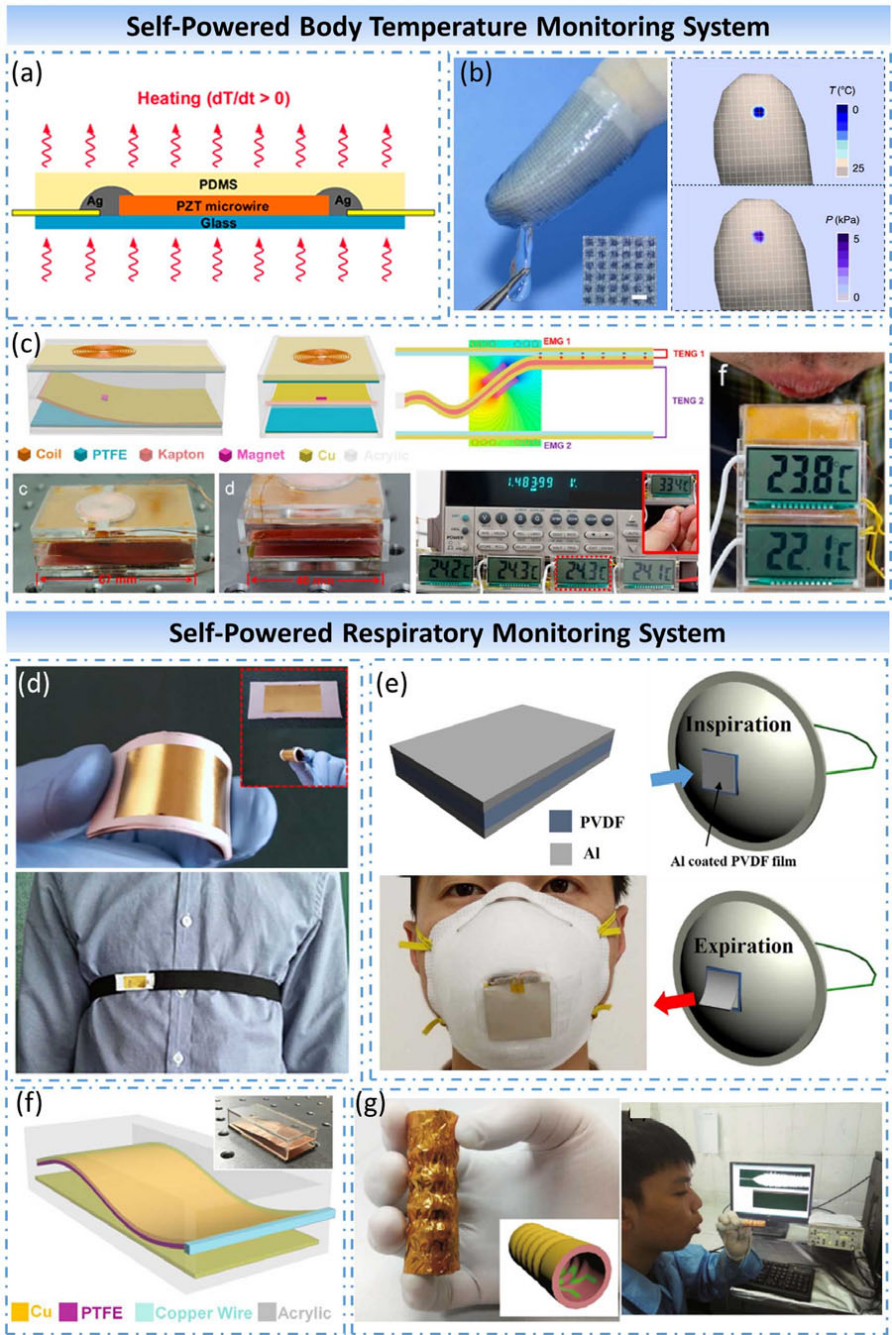
Self-powered body temperature monitor system and self-powered respiratory monitor system. (a) Self-powered temperature sensor based on a PyNG. (b) Self-powered temperature–pressure dual-parameter sensor fabricated by organic thermoelectric materials. (c) Wireless temperature sensor system based on hybridized nanogenerator. (d) Wearable self-powered active sensor for respiration monitoring based on a flexible piezoelectric nanogenerator. (e) Wearable respiration sensor based on a PyNG integrated with an N95 respirator. (f) Air-flow-driven triboelectric nanogenerator used for monitoring both breathing status and exhalation volume. (g) Self-powered exhaled breath analyzer based on a PANI/PVDF piezo-gas-sensing array.

In 2012, Yang et al. presented a pyroelectric nanogenerator (PyNG) as a self-powered temperature sensor, which is fabricated by a single lead zirconate titanate (PZT) micro/nanowire (Yang et al., 2012) (Figure 6a). The output voltage of PZT nanowire has a positive correlation with the rate of temperature change, and the minimum detection limit of temperature change is 0.4 K at room temperature, which can be used to detect the temperature of human fingertips. In 2015, Zhang et al. reported a flexible and self-powered temperature–pressure dual-parameter sensor, which was fabricated by organic thermoelectric materials and a microstructured supporting frame (Zhang et al., 2015) (Figure 6b). The self-powered dual-parameter sensor can distinguish temperature changes less than 0.1 K and pressure changes up to 28.9 kPa^−1^ simultaneously based on the coupling of thermoelectric and piezoresistive mechanisms. Furthermore, highly integrated wearable sensor arrays for electronic fingers were also developed by inkjet printing. In 2015, Wang et al. designed a hybridized nanogenerator by integrated two TENGs and two EMGs in a small acrylic box expertly (Wang et al., 2015) (Figure 6c). The hybridized nanogenerator can sustainably drive four commercial temperature sensors simultaneously by collecting the mechanical energy of air-flow. Under an air-flow speed of about 18 ms^−1^, the hybridized nanogenerator can deliver largest output powers of 3.5 mW for one TENG at a loading resistance of 3 MΩ and 1.8 mW for one EMG at a loading resistance of 2 kΩ, respectively. A wireless temperature sensor was also successfully driven, and emitted temperature date by a hybridized nanogenerator demonstrated its potential for self-powered wireless temperature sensing networks.

##### 5.1.1.3. Self-powered respiration monitor system

Respiration is another critical indicator of human life. Respiratory motion is inherently associated with all life activities of the human body, and it can also reflect the health condition of a person. Sleep apnea-hypopnea syndrome is a common respiratory disorder in clinical practice that has a high potential risk. Sleep monitoring is currently the gold standard for the clinical diagnosis of this disease. Therefore, it is crucial and urgent for sleep medicine to develop a respiratory monitoring device that can continuously monitor spontaneous breathing for a long time without affecting the quality of sleep (van Loon et al., 2015). Also, some studies have shown that the gas exhaled by a person contains a variety of chemical components, which can also be used to detect the health status of the human body, and even suggestive diagnosis of some specific diseases. As a consequence, the detection and analysis of exhaled air are equally important besides the monitoring of respiratory movements.

In 2017, Liu et al. presented a wearable self-powered active sensor for respiration monitoring, which was fabricated by an electrospinning poly(vinylidene fluoride) thin film on the silicon substrate after polarized (Liu et al., 2017) (Figure 6d). Through integrated with an elastic strap, this flexible PENG can monitor the human’s breathing in real-time by converting the mechanical energy of breathing movement into electrical signals. In 2017, Xue et al. proposed a wearable PyNG by integrated a PVDF film with an N95 respirator (Xue et al., 2017) (Figure 6e). Utilizing the temperature fluctuation that takes place when human exhale and inhale, the output signals of this wearable PyNG can reflect the breathing state of the wearer through the pyroelectric effect of PVDF. The maximal power of this PyNG can reach up to 8.31 μW with an external load of 50 MΩ. In 2018, Wang et al. developed an air-flow-driven TENG based on a flexible n-PTFE thin film and its oscillation under laminar air flow (Wang et al., 2018a) (Figure 6f). This n-PTFE TENG can convert the mechanical energy of the air flow into electrical energy, and the electrical signal is under a significant correlation with the air flow rate. Under the action of breathing, the maximum output power of the TENG reached 1.3 mW at a load resistance of 15.1 MΩ. When integrated with a mask, this TENG can be used to monitor both breathing status and exhalation volume. In 2018, Fu et al. fabricated a self-powered exhaled breath analyzer based on a PANI/PVDF piezo-gas-sensing array (Fu et al., 2018) (Figure 6g). Combined with the piezoelectric effect of PVDF and the gas-sensing properties of PANI electrodes, five different sensing units can detect the gas concentration of different gas markers contained in human exhaled gas with no needing for external power supply. This idea is expected to be a potential diagnostic approach for some specific diseases.

##### 5.1.1.4. Self-powered pulse monitor system

The heart is the power that the blood pumps out, and it also guarantees the normal functioning of the various organ systems and the entire body. Every pulse of a person represents a forceful heartbeat, and the number of heartbeats per minute is the heart rate. The heart rate is the most unequivocal sign of a person’s heart health. There are numerous clinical diseases, especially heart disease that can change the pulse of a person. For people at high risk of cardiovascular disease, long-term continuous monitoring of heart rate or pulse can establish the first line of prevention besides hospital inspections, discover the abnormal cardiac activity in time and help the diagnosis and prevention of cardiovascular-related diseases. However, the electrocardiograph (ECG)/pulse monitoring devices currently used in clinical practice are either bulky, inconvenient to carry and move, or have short battery life and low accuracy. Aim to achieve long-term continuous pulse monitoring as well as ensuring the high accuracy of monitoring results, many research teams have developed self-powered pulse sensors with high sensitivity and portability, which is of great significance for promoting mobile health and telemedicine.

**Figure 7.**
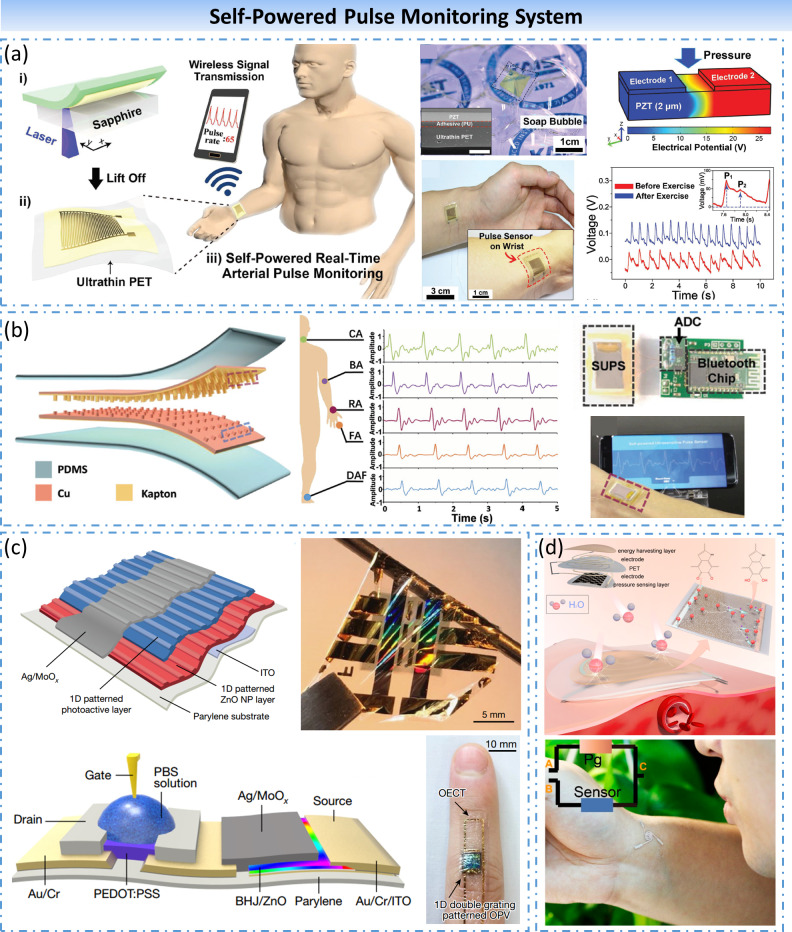
Self-powered pulse monitoring system. (a) Ultrathin epidermal piezoelectric sensor for real-time arterial pulse monitoring. (b) Flexible self-powered ultrasensitive pulse sensor based on triboelectric effect. (c) Self-powered ultra-flexible biosensor based on nanograting-patterned organic photovoltaics. (d) Moisture-driven flexible multifunctional sensing system based on flexible piezoresistive sensor and moisture-enabled power generator.

In 2017, Park et al. presented a self-powered ultrathin epidermal piezoelectric sensor for real-time arterial pulse monitoring (Park et al., 2017) (Figure 7a). By means of an inorganic-based laser lift-off technique, a PZT thin film was transferred onto an ultrathin PET (Polyethylene terephthalate) substrate, which endows the flexible sensor with a sensitivity of 0.018 kPa^−1^, a response time of 60 ms, and provides the ability to monitor tiny radial/carotid arterial pulse through rugged human epidermis. Furthermore, pulse signals gathered by the piezoelectric sensor wirelessly transmitted to a smartphone via Bluetooth was also demonstrated. In 2017, Ouyang et al. developed a flexible self-powered ultrasensitive pulse sensor (SUPS) based on the triboelectric effect (Ouyang et al., 2017) (Figure 7b). The SUPS can directly produce an output of up to 1.52 V when detecting human pulses, the response time is 50 μs, and the peak signal-to-noise ratio is as high as 45 dB. Notably, the pulse wave signal captured by SUPS is consistent with the second derivative of the conventional pulse signal, which can be utilized for indicative diagnosis and antidiastole to some specific cardiovascular diseases such as arrhythmia and atrial fibrillation after characteristic exponent analysis. In 2018, Park et al. demonstrated a self-powered ultra-flexible biosensor based on nanograting-patterned organic photovoltaics, which enables real-time accurate monitoring of heart rate (Park et al., 2018) (Figure 7c). The biosensor was fabricated by integrating OPV (organic photovoltaic power sources) with OECTs (organic electrochemical transistors) on an ultra-thin parylene substrate (thickness of 1 μm). Such OECTs can operate under low voltage conditions of about 1 V, and OPV can be fully consistent with the functional requirements, even under normal room light conditions. When the biosensor attached to a finger and the gel electrode connected to a person’s chest, OECTs can acquire clear and stable heart rate signals, with a power-conversion efficiency (PCE; 10.49%) superior to that of other flexible OPVs, resulting in a high power-per-weight (11.46 W/g). In 2019, Li et al. designed a novel moisture-driven flexible multifunctional sensing system and his system was prepared by connecting a flexible piezoresistive sensor in series with a moisture-enabled power generator based on a porous polydopamine layer with a hydroxy group gradient (Li et al., 2019) (Figure 7d). The environmental-moisture-induced self-powered sensing system is under a high sensitivity to both humidity and pressure, which can provide real-time monitoring of human physiological signals such as breathing and pulse by collecting ambient moisture and converting into electricity.

#### 5.1.2. Self-powered wearable therapy system

In addition to diagnosing the physical condition of the subject, a complete course of treatment should also be able to achieve the steps of therapy. Wearable therapy systems often require long-time treatment, and battery life grows up to be a challenge. Therefore, the Self-powered wearable therapy system demonstrates its advantages in long-time treatment. Self-powered wearable therapy system’s research content includes drug delivery, tissue repair, electrical stimulation and so on.

##### 5.1.2.1. Self-powered drug delivery system

“Smart Medicine” and “Precision Medicine” are two major trends in the future development of medical care. The precise release and slow-release control of drugs can significantly increase the targeting of drugs, and at the same time, can significantly increase the compliance of patients or reduce the side effects of drugs. It is another important research direction and goal of the clinical treatment of tumors. The self-powered drug delivery system (DDS) can actively regulate the release amount and release the target of the drug according to the need of the human body and form a closed-loop system. In recent years, with the rapid development of sustainable energy and self-powered systems, researchers have been developing a variety of self-powered DDSs.

**Figure 8.**
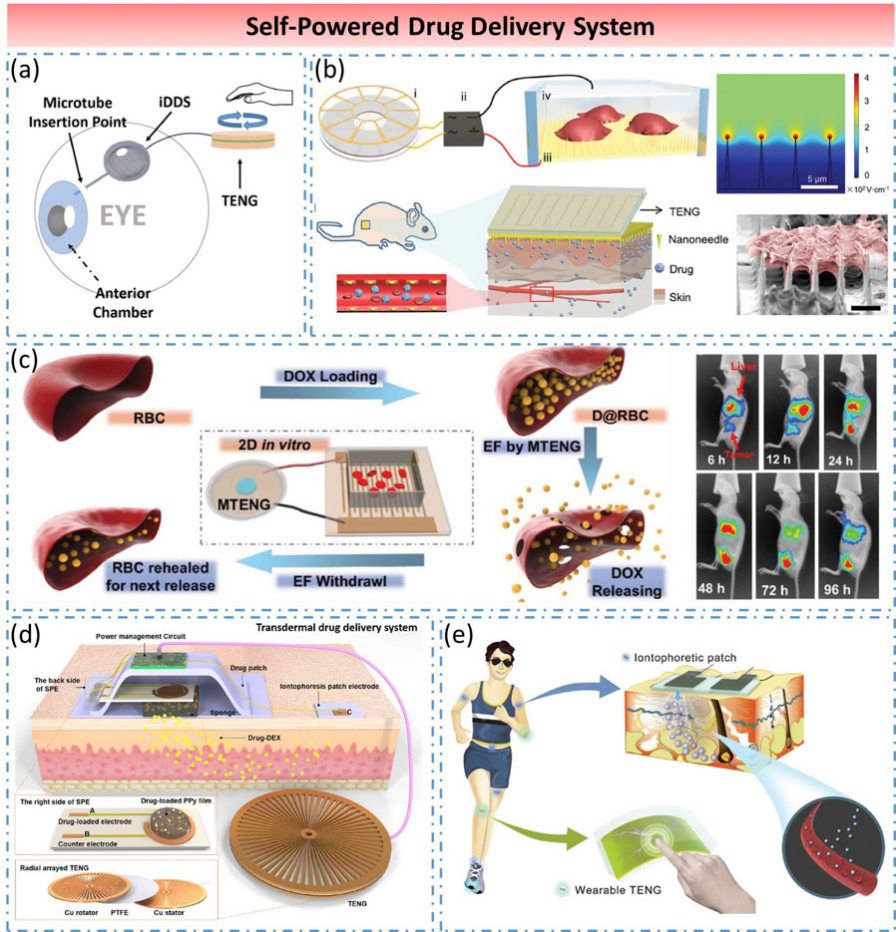
Self-powered drug delivery system. (a) Self-powered trans-sclera drug delivery system. (b) Self-powered electroporation intracellular drug delivery system. (c) Self-powered controllable drug delivery system for highly efficient in vivo cancer therapy. (d) Self-powered, on-demand transdermal drug delivery system. (e) Self-powered wearable iontophoretic transdermal drug delivery system.

In 2017, Song et al. demonstrated the first TENG-based self-powered drug-delivery system (Song et al., 2017) (Figure 8a). The system consisted of an electrochemical microfluidic pump and a rotation type TENG. When human hands rotated TENG, the aqueous solution in a microfluidic pump was electrolyzed to generate gas, which caused the drug to be slowly released from the drug reservoir. The ex vivo trans-sclera drug delivery in porcine eyes was also demonstrated to verify the functionality of the system for ocular drug delivery. In 2019, Liu et al. presented a self-powered intracellular DDS with high efficiency and minimal cell damage in vitro and in vivo (Liu et al., 2019b) (Figure 8b). The system was composed of a stable voltage pulse source based on a biomechanical-energy-powered TENG and a silicon nanoneedle-array electrode for minimizing cellular damage during electroporation. The self-powered electroporation system effectively delivered exogenous materials into different types of cells, indicated its great potential for self-tuning drug delivery and wearable medicine.

In 2019, Zhao et al. realized the first nanogenerator supported controllable DDS for cancer therapy (Zhao et al., 2019) (Figure 8c). A magnet-TENG (MTENG) was fabricated to combine with the antitumor DDS based on doxorubicin-(DOX-) loaded red blood cells. The MTENG shows a strong power generation capacity. Its power density can reach 100 mW/m^2^ and this huge electric pulse can generate a periodic electric field. Under the electric field stimulation of MTENG, the system enabled a controllable drug releasing at the tumor site. Furthermore, the system achieved prominent tumor treatment effects under a low drug dosage at three levels: a 2D culture of HeLa cells, 3D multicellular spheroids, and tumor-bearing nude mice, which demonstrated a promising approach for cancer therapy in the clinic. In 2019, Ouyang et al. proposed a self-powered, on-demand transdermal DDS, which consisted of a miniaturized rotary TENG, a power management circuit and transdermal patches (including drug patch and iontophoresis patch electrode) (Ouyang et al., 2019) (Figure 8d). The system can achieve an actively adjustable drug release rate from 0.05 to 0.25 μg/cm^2^ per minute and improve the efficiency of transdermal drug delivery (TDD) by ∼50%, indicating a prospect of wearable drug delivery device for personalized healthcare. In 2019, Wu et al. designed a self-powered wearable iontophoretic TDD system for closed-loop sensing and treatment by utilizing biomechanical energy (Wu et al., 2019) (Figure 8e). The system included a hydrogel-based soft patch with side-by-side electrodes to realize noninvasive iontophoretic TDD and a wearable TENG to convert electricity for iontophoresis. The feasibility of the system was manifested in the transdermal delivery of the simulated drugs (fluorescent dyes) into a piece of pigskin.

##### 5.1.2.2. Self-powered tissue repair system

Tissue repair and regenerative medicine is at the center of attention and research hotspot in the world today, involving almost all areas of personal injury, disease treatment, and antiaging. Through applying extra stimulation to activate the self-healing ability of the human body fully, damaged tissues or organs can be regenerated and reconstructed, and biological functions can be restored and enhanced even to achieve permanent rehabilitation. It has been one of the most critical directions in contemporary regenerative medicine research. A new type of treatment for tissue repairing is converting the human body’s energy into light, electricity, thermal energy, and so on, and applying it to the damaged part of the human body. The type of wearable therapy device without the requirement of external power supply has brought new opportunities to the field of tissue repair and regenerative medicine.

**Figure 9.**
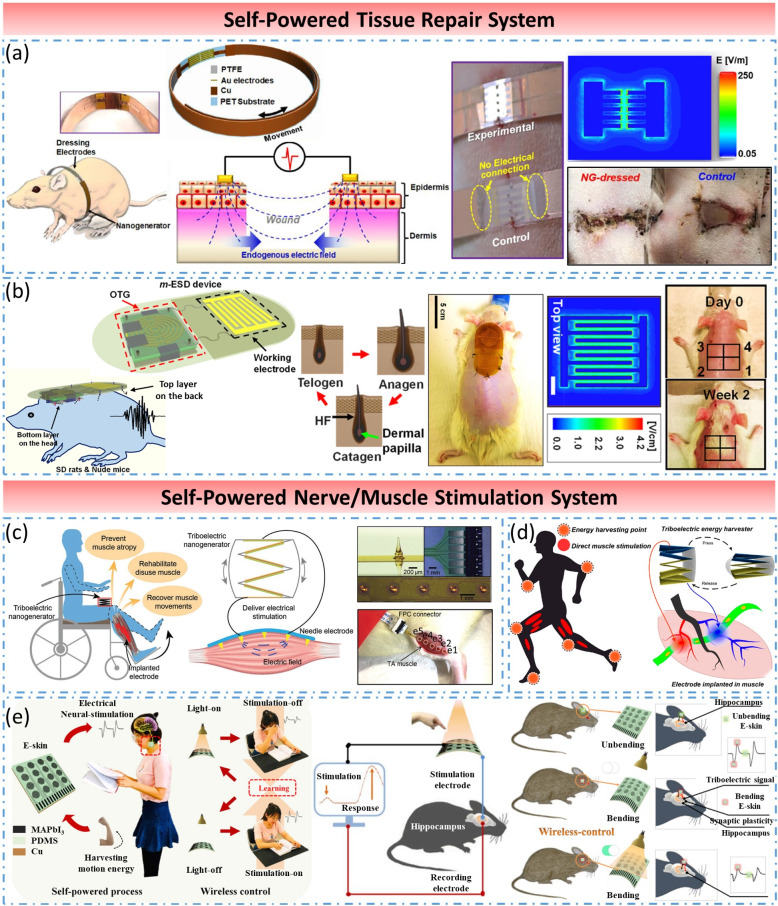
Self-powered tissue repair system and self-powered nerve/muscle stimulation system. (a) Self-powered electrical bandage for accelerating skin wound healing. (b) Self-activated wearable electric stimulation device for effective hair regeneration. (c) and (d) Self-powered TENG-based system for direct muscle stimulation. (e) Self-powered photo-operate neural-stimulating e-skin for characterization of synaptic plasticity.

In 2015, Tang et al. proposed a self-powered low-level laser cure system for osteogenesis (Tang et al., 2015). The system was fabricated by integrating an infrared laser irradiation unit and a TENG. Under the influence of the system, the proliferation and differentiation of mouse embryonic osteoblasts were significantly accelerated, and the increment of osteoblast mineralization was also pointed out. Moreover, the infrared laser can be successfully excited by walking when applied to the system on the human body, showing a portable clinical potential cure method for bone remodeling or orthodontic treatment. In 2018, Long et al. developed a self-powered electrical bandage based on a wearable nanogenerator, which can accelerate skin wound healing efficiently by converting kinetic energy from skin movements into electrical stimulus signals (Long et al., 2018) (Figure 9a). Animal experiments showed an amazing result that the wounds originally took 12 days to heal were completely healed within 3 days under the stimulation of a wearable nanogenerator. This self-powered electrical bandage showed great potential in enhancing skin regeneration, which can provide a straightforward treatment strategy for some difficult-to-heal skin wounds. In 2019, Yao et al. presented a self-activated wearable electric stimulation device for effective hair regeneration (Yao et al., 2019) (Figure 9b). A wearable omnidirectional pulse generator was designed to collect the kinetic energy of the head in any direction and convert into an AC electric field to load on the interdigitated electrode connected with a generator. Under the influence of this device, rattus *norregicus*s, and nude mice both achieved significant results in promoting hair regeneration, which indicated a promising hair regeneration strategy utilizing a SWE.

##### 5.1.2.3. Self-powered nerve/muscle stimulation system

Electrical stimulation has long served in rehabilitation therapy. In the beginning, passive stimulation was used to delay muscle atrophy caused by muscle disuse, diminish inflammation, ease pain, and promote physical recovery in hemiplegia patients. Nowadays, there are various types of muscle stimulation and nerve stimulation, which can replace or correct the lost functions of limbs and organs. To this day, electrical stimulation is increasingly accepted and recognized by people and has also become an important research direction for the clinical treatment of neuratrophia and amyotrophia. Traditional electrical stimulation devices are bulky and usually require professional medical staff to operate them, which greatly limits the time and space for treatment. Fortunately, the emergence of self-powered systems has made wearable, sustainable electrical stimulation devices possible. In 2019, Wang et al. presented a self-powered system for direct muscle stimulation based on a multiple-channel spiked epimysial electrode and a stacked-layered TENG (Hong et al., 2019) (Figure 9c). In order to guarantee the feasibility of the system, two key issues of TENG direct muscle stimulation were fully studied, which improved the efficiency and stability of TENG muscle stimulation. In the same year, they also verified that the electrode-motoneuron position and the stimulation waveform polarity of TENG severely affect the stimulation efficiency (Wang et al., 2019a) (Figure 9d). These studies are of great significance to the practical use of a self-powered system-based TENG for rehabilitation treatment of muscle function loss. In 2020, Guan et al. demonstrated a self-powered photo-operate neural-stimulating e-skin for characterization of synaptic plasticity (Guan et al., 2020) (Figure 9e). The e-skin was fabricated by flexible MAPbI3/PDMS units based on photodetecting/triboelectric coupling effect, which can generate electrical output nerve stimulation signals by human body activities and regulated by photo illumination. Through the self-powered neural-stimulating skin, the brain activities in CA3 area of mouse hippocampus was successfully evoked, and the field excitatory postsynaptic potentials were recorded in CA1 area of the mouse hippocampus, indicating that the self-powered e-skin is an effective approach for quantification of neural plasticity changes and a promising battery-free neural stimulating system.

### 5.2. Self-powered wearable intelligent system

Except for applications in medical science, SWE has also application prospects in the fields of robotics and smart wear. Self-powered wearable intelligent systems could act as the bridge for information interaction between humans and machines.

#### 5.2.1. Self-powered motion information detecting system

**Figure 10.**
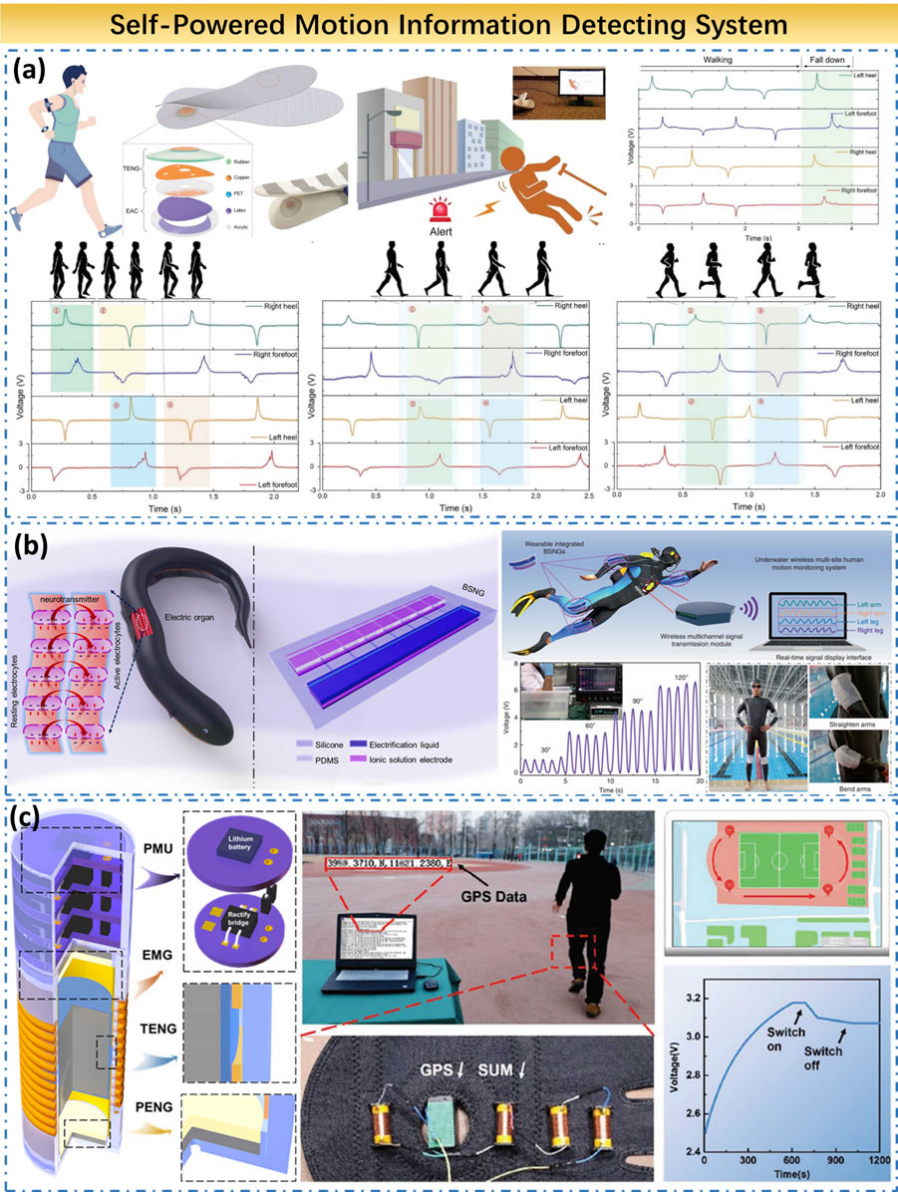
Self-powered motion information detecting system. (a) Multifunctional gait monitoring system based on triboelectric nanogenerator. (b) Underwater wireless multisite motion monitoring system based on bionic stretchable nanogenerators. (c) Self-powered GPS location system based on battery-like self-charge universal modules.

The SWEs are used as motion monitoring systems. Its main research object, including the elderly, patients, athletes, and other people who need to monitor their motion conditions. The main research contents include the gait, motion state, current position, and other information about the wearer. Among them, the gait of the human body is a significant indicator for assessing the incidence of the elderly or the injured. Yang et al. developed a smart insole based on TENG to determine whether a person is in walking, jumping, running, or stationary state by collecting different mechanical signals generated by the human body on the soles (Figure 10a). Besides, this system can also respond to abnormal signals generated during a fall, and produce a timely alarm for the elderly’s abnormal fall. This system has high durability, fast response time, and excellent mechanical stability. As a typical SWE-based motion detection system, it is worthy of reference for subsequent researchers (Lin et al., 2019).

A significant problem of SWEs accounts for waterproofing. When used in motion monitoring, it is often accompanied by the discharge of a large amount of sweat from the human body, which puts forward requirements for the waterproof performance of the device. In particular, in some sport, such as diving and swimming, the sport scenes of the athletes are completely underwater, and higher requirements for waterproof performance are required for the underwater motion monitoring of the athletes. In response to this problem, Li et al. developed an underwater motion monitoring system based on bionic electricity-generating cells (Figure 10b). It uses the coupling effect of frictional electrification and electrostatic induction to collect the electrical signals generated by the limbs of the athlete during exercise. It then sends the obtained signal to the shore receiving device through a Bluetooth wireless transmitting device to analyze the underwater posture of the athlete in real-time. The power density of this system can reach 18 mW/m^2^ and it will help in swimming posture correction and targeted training. However, the miniaturization and intelligence of the device have yet to be improved. Also, this work borrows structurally from the structure of electric eel power generating cells, which is an important application of bionics in the field of SWE and has a great inspiration for the development of future SWE devices (Zou et al., 2019).

Besides the various mechanical information generated during human motion, the position information of the subject is also essential. The previous work collected mechanics Information belongs to the sensor type SWE. To monitor the position information, it is necessary to gather the energy generated by the wearer’s motion and then provide energy for the position electronics, which belongs to the category of energy type SWE. Li et al. designed a self-charge universal module, which used three types of generators to generate electricity (Figure 10c). By collecting the mechanical energy generated by the subject’s 10-min jog, the self-charging universal module can charge the battery in the GPS (Global Positioning System) to 3.2 V, in which power density is 2023 μW/g. This energy can drive the GPS to work for more than half an hour and complete positioning, showing its great potential as an emergency power source. Here, the use of hybrid generators to collect electrical energy can also have many advantages, enhancing the power of the generator and increasing the applicable range under different conditions when mechanical energy is collected (Tan et al., 2019).

#### 5.2.2. Self-powered man–machine interactive system

Commonly, materials used in SWE are stable and easy to process. The structure is generally relatively simple, and it is convenient to make arrays and integrate, which makes it very suitable for application to human–machine interaction systems. Human–machine interaction refers to the exchange of information between man and machine. The machine collects information through the human–machine interface and visualizes the information. Human beings send out a command to the machine by a human–machine interface and achieve real-time control of the machine.

**Figure 11.**
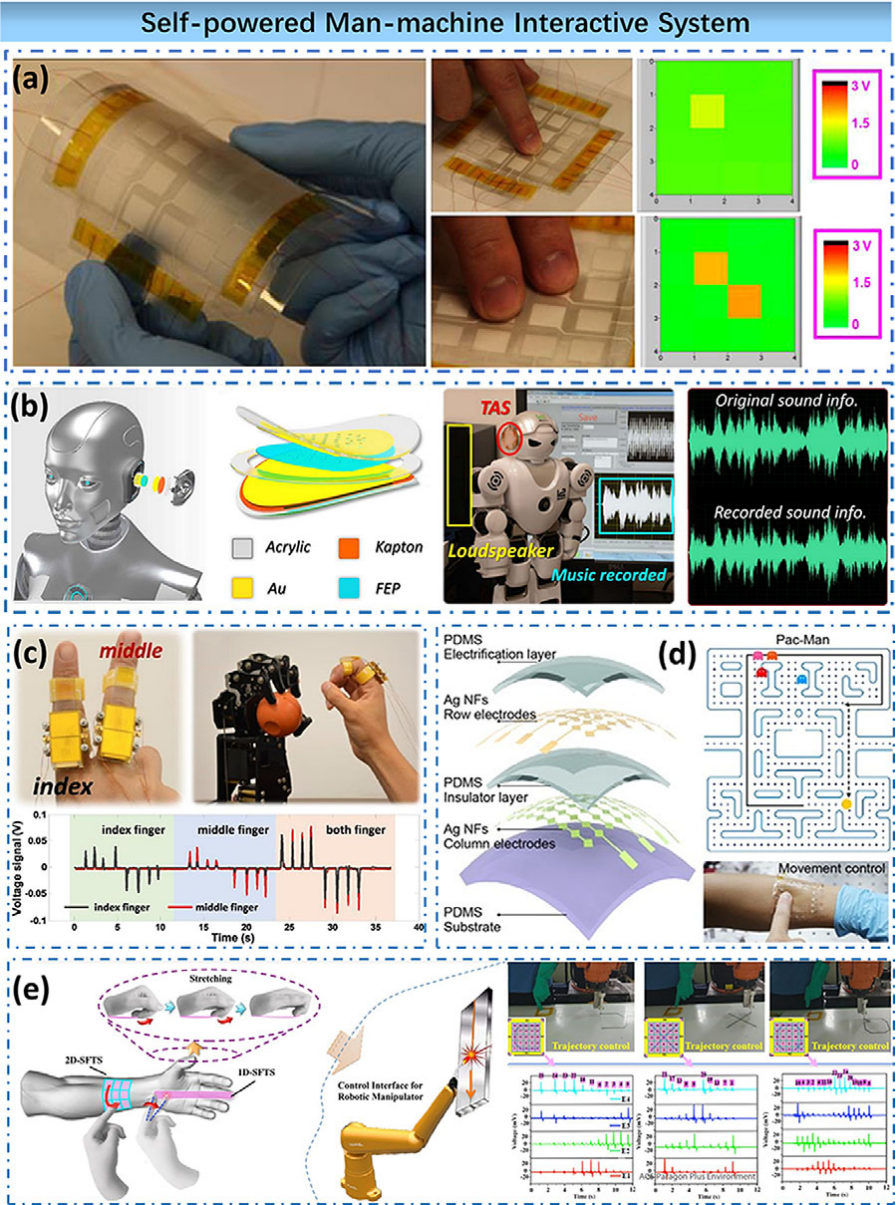
Self-powered man–machine interactive system. (a) Self-powered tactile sensing panel. (b) Self-powered triboelectric auditory sensor for social robotics and hearing aids. (c) Rotation sensing and gesture control of a robot joint via triboelectric quantization sensor. (d) Self-powered triboelectric tactile sensor with metallized nanofibers for wearable electronics. (e) Self-powered wearable flexible patch as 3D motion control interface for robotic manipulator.

Yang et al. developed an arrayed electronic skin system with excellent features such as flexibility, transparency, and water resistance (Figure 11a). The designed sensor array can recognize the protrusions of distinct patterns based on the principle of the TENG to form artificial tactile. For single pixel, the open circuit voltage can reach 1.613 V and a short circuit current density is 47.308 mA/m^2^ under the pressure of 612.5 kPa (Yang et al., 2013). Hu et al. designed an auditory sensor to convert acoustic information into mechanical information, and then converted mechanical information into electrical information for identification based on the principle of TENG (Figure 11b). TENG’s recognition of mechanical information has the characteristics of wide frequency, which can cover the sound frequency of people’s daily communication (Guo et al., 2018).

Another critical application of the human–machine interactive system is mechanical control. By setting sensors on the joints of the wearer, SWE could detect the movement of different joints, which is a feasible idea to control the robot in real-time. Hu et al. designed a robotic arm synchronous control system (Figure 11c). The TENG is placed at the finger joint, and the researcher’s finger movement could control the robot arm. This method can realize real-time high-precision robotic control (Pu et al., 2018).

In 2018, Wang et al. developed a transparent, stretchable tactile sensor based on the TENG principle (Figure 11d). The PVA in the device is made by electrospinning. Combined with wet etching and other processes, an 8 × 8 array is made. The device has both high transparency, high pressure sensitivity, stretchability, and multitouch operation. It can simultaneously achieve functions such as biomechanical energy collection and tactile sensing, providing a new perspective for the preparation of transparent, stretchable tactile sensors (Wang et al., 2018b). In addition to move on a plane, a real mechanical arm sometimes needs to move in a vertical direction. For this reason, a new operation interface is required to control the vertical movement of the robot arm. Li et al. used a hydrogel, PDMS, silica gel, and other materials to ensure that a three-dimensional robotic arm control system (Figure 11e). Among them, the control of movement in the plane direction is realized by a two-dimensional sensor array. An additional one-dimensional sensor patch is set to control the perpendicular direction. This mechanical control system can detect the continuous sliding information of finger motion, including the speed, acceleration, and path of finger motion. At the same time, the path is reflected in the manipulation of the robot arm.

## 6. Conclusion and Outlook

In recent years, research on SWE has developed rapidly. Scholars have carried out various researches on SWE. The research directions are primarily reflected in the improvement of performance, the expansion of application scenarios, the combination with new technologies, new structures, and new principles. However, there are still many key issues that need to be resolved if SWE wants to truly change from laboratory research to products that meet the requirements of people’s daily use, which deserves our in-depth exploration.

The first is miniaturization. At present, with the development and maturity of semiconductor technology, the size of electronics has become small enough. However, the current SWE technology is still in the research stage of the laboratory. With the industrialization of SWE, its precision and miniaturization will be significantly improved. The second problem is the durability of SWE. The mechanical durability of the device has to be tested in a complex environment. In addition, for energy type SWE, higher battery capacity, and energy conversion efficiency are necessary. Improvements in battery capacity, energy conversion capabilities, and so on, can improve the productive working hours of SWE. In the future, the improvement of battery capacity and PCE may depend on the choice of materials and advances in power generation technology. The third issue is integration. The sole function SWE can no longer meet the needs of users. The integration of multiple functions will become a development trend. The integration of various functional modules increases the design difficulty, and at the same time, it also puts forward higher requirements for energy consumption. The final challenge is the intelligence of SWE. Current SWE research often transfers the collected information to the computer for processing and analysis, which dramatically reduces the advantage that SWE can be carried freely. Real-time on-site acquisition and real-time on-site analysis are the directions of future SWE development.

To achieve SWE’s miniaturization, durability, integration, and intelligence, the most important challenge is the further discovery of materials science. The vigorous development of the microelectromechanical system (MEMS) field has given SWE system great inspiration. However, SWE’s flexibility requirements make existing MEMS technology less applicable. Especially research on micro flexible circuits, although many researchers have paid enough attention to this research direction, the existing micro flexible circuit technology is still far from the actual user requirements. In order to realize a truly effective flexible circuit, researchers have developed various ideas. Such as functional hydrogel (Liu et al., 2020), conductive polymer (Oh et al., 2016), ionic conductor (Kim et al., 2020) and so on. These research directions being studied may become a major breakthrough point in the development of SWE. Another area where fundamental breakthroughs are needed is the energy harvesting efficiency of SWE. The existing energy harvesting technology is theoretically sufficient, but in the actual application process, due to the attenuation caused by many factors, the actual effect is unsatisfactory. Multilayer, array, and hybrid generator are possible solutions to improve generation efficiency.

**Figure 12.**
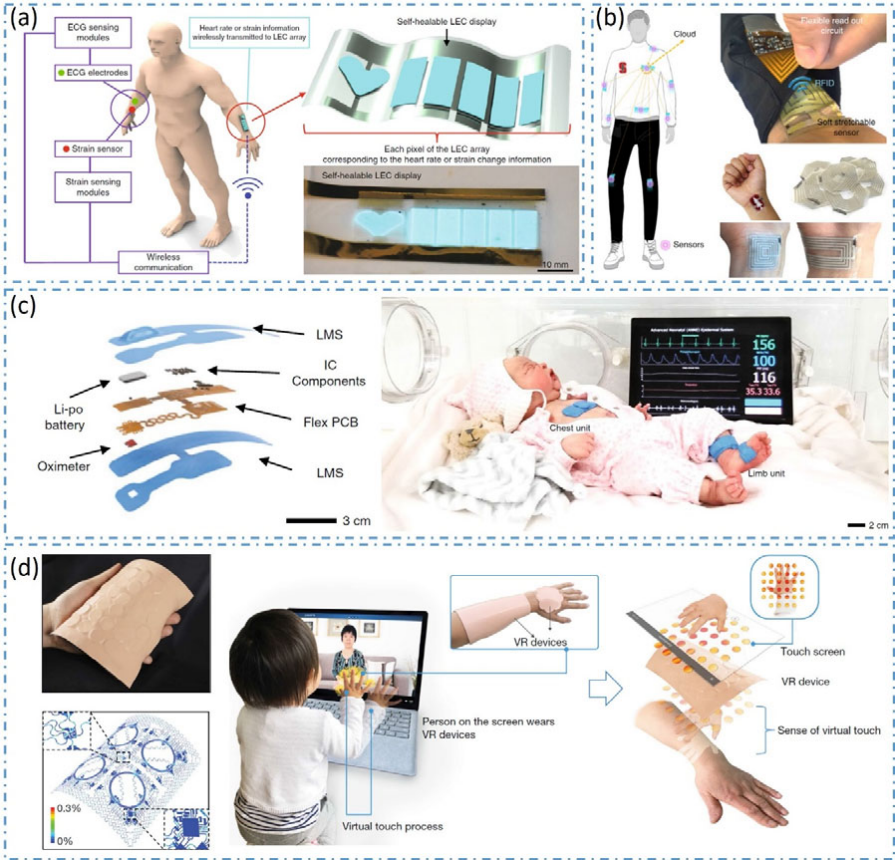
Future development trends of wearable electronics. (a) Integrated self-healable electronic skin system. (b) Wireless body area sensor network system. (c) Wireless skin-interfaced biosensors for newborn physiological monitoring. (d) Skin-integrated wireless haptic interfaces.

Some laboratories focus on the work of wearable electronic products. Strictly speaking, part of their work is not within the scope of SWE. But these works provide promising solutions for solving several challenges of SWE. In this regard, there are many internationally renowned research teams that have been working hard to explore and have made a lot of important research work. For example, Professor Bao Zhenan’s research team has long been devoted to flexible electronics and electronic skins. Through efforts in new materials science, traditional wearable electronics are endowed with a stretchable, self-healing, wireless, and visual integrated system (Donghee et al., 2018) (Figure 12a). On the way to seeking new signals and energy transmission method, a wireless body area sensor network system was proposed. The on-skin wireless sensor of the system was battery-free and chip-free, working like an ID card, using radio frequency identification technology to absorb energy from the clothing receiver, then read the data from the skin and send it back to the receiver (Niu et al., 2019) (Figure 12b). Professor John A. Rogers’ research team has been exploring the field of flexible electronics for decades. The continuous innovation of materials and device structures and the continuous improvement of processing technology make their products available for clinical diagnosis and monitoring. Recently, they developed a wireless, nonintrusive skin-interfaced biosensing system for neonatal and pediatric intensive care (Ha et al., 2020) (Figure 12c). The system can provide measurements of heart rate, respiration rate, temperature, and blood oxygenation comparable to existing clinical standards without any adverse effects on the baby’s skin. In addition to medical applications, they are also trying to develop some promising technology to change people’s daily lives. Recently, for the first time, they realized the integration of complex touch sense into virtual and augmented reality. They developed a wireless, battery-free electronic system platform and tactile interfaces, which can be gently attached to the skin and transmit tactile information remotely through programmable localized mechanical vibrations (Yu et al., 2019) (Figure 12d).

SWE has received extensive attention and research due to its portability, wide application space, and high-value future market. In the future, the combination of materials with new features and new functions, wireless, passive, soft and easy to wear, good safety and stability, facing practical clinical and market applications, will be the development direction of next generation of wearable electronics. Meanwhile, the hybridization of these new technologies and self-powered system, will further promote the development of SWE.
